# Influence of midgut microbiota in *Anopheles stephensi* on *Plasmodium berghei* infections

**DOI:** 10.1186/s12936-018-2535-7

**Published:** 2018-10-25

**Authors:** Devaiah Monnanda Kalappa, Pradeep Annamalai Subramani, Sowmya Kanchanahalli Basavanna, Susanta Kumar Ghosh, Varadharajan Sundaramurthy, Sreehari Uragayala, Satyanarayan Tiwari, Anupkumar R. Anvikar, Neena Valecha

**Affiliations:** 1 ICMR-National Institute of Malaria Research (Field Unit), Nirmal Bhawan-ICMR Campus, Poojanahalli, Kannamangala Post, Devanahalli Taluk, Bengaluru, Karnataka 562110 India; 2 Manipal Academy of Higher Education, Manipal, Karnataka 576104 India; 3 National Centre for Biological Sciences, Tata Institute of Fundamental Research, GKVK Campus, Bellary Road, Bengaluru, 560065 India; 4 ICMR-National Institute of Malaria Research, Sector 8, Dwarka, New Delhi 110077 India

**Keywords:** Malaria, *Anopheles*, Mosquitoes, Microbiota, *Plasmodium berghei*, *Veillonella*

## Abstract

**Background:**

The native gut microbiota of *Anopheles* mosquitoes is known to play a key role in the physiological function of its host. Interestingly, this microbiota can also influence the development of *Plasmodium* in its host mosquitoes. In recent years, much interest has been shown in the employment of gut symbionts derived from vectors in the control of vector-borne disease transmission. In this study, the midgut microbial diversity has been characterized among laboratory-reared adult *Anopheles stephensi* mosquitoes, from the colony created by rearing progeny of wild-caught mosquitoes (obtained from three different locations in southern India) for multiple generations, using 16S ribosomal RNA (rRNA) gene sequencing approach. Further, the influence of native midgut microbiota of mosquitoes on the development of rodent malaria parasite *Plasmodium berghei* in its host has been studied.

**Methods:**

The microbial diversity associated with the midgut of *An. stephensi* mosquitoes was studied by sequencing V3 region of 16S ribosomal RNA (rRNA) gene. The influence of native midgut microbiota of *An. stephensi* mosquitoes on the susceptibility of the mosquitoes to rodent malaria parasite *P. berghei* was studied by comparing the intensity and prevalence of *P. berghei* infection among the antibiotic treated and untreated cohorts of mosquitoes.

**Results:**

The analysis of bacterial diversity from the midguts of *An. stephensi* showed *Proteobacteria* as the most dominant population among the three laboratory-reared strains of *An. stephensi* studied. Major genera identified among these mosquito strains were *Acinetobacter*, *Pseudomonas*, *Prevotella*, *Corynebacterium*, *Veillonella*, and *Bacillus*. The mosquito infectivity studies carried out to determine the implication of total midgut microbiota on *P*. *berghei* infection showed that mosquitoes whose native microbiota cleared with antibiotics had increased susceptibility to *P. berghei* infection compared to the antibiotic untreated mosquitoes with its natural native microbiota.

**Conclusions:**

The use of microbial symbiont to reduce the competence of vectors involved in disease transmission has gained much importance in recent years as an emerging alternative approach towards disease control. In this context, the present study was aimed to identify the midgut microbiota composition of *An. stephensi*, and its effect on the development of *P. berghei*. Interestingly, the analysis of midgut microbiota from *An. stephensi* revealed the presence of genus *Veillonella* in *Anopheles* species for the first time. Importantly, the study also revealed the negative influence of total midgut microbiota on the development of *P. berghei* in three laboratory strains of *An. stephensi*, emphasizing the importance of understanding the gut microbiota in malaria vectors, and its relationship with parasite development in designing strategies to control malaria transmission.

**Electronic supplementary material:**

The online version of this article (10.1186/s12936-018-2535-7) contains supplementary material, which is available to authorized users.

## Background

Malaria is caused by the parasite of genus *Plasmodium* and transmitted by female *Anopheles* mosquitoes. Five *Plasmodium* species are capable of infecting human host, namely *Plasmodium falciparum*, *Plasmodium vivax*, *Plasmodium ovale*, *Plasmodium malariae*, and *Plasmodium knowlesi*. Malaria is considered as an important public health burden, especially in sub-Saharan Africa, with an estimated 216 million cases reported worldwide in 2016 [1, 2]. Malarial parasites have a complex life cycle, which alternates between the vector mosquito and the vertebrate host [2]. Prior to successful transmission of infective sporozoites to the vertebrate host, the parasite has to undergo a series of developmental transitions within the mosquito [3]. The sexual stage development of the parasite within the mosquito begins with the ingestion of gametocytes during an infectious blood meal. In the mosquito gut, various physiological and environmental factors, together with the presence of xanthurenic acid, trigger gametogenesis, where the male gametocytes undergo the process of exflagellation producing microgametes and female gametocytes to form macrogametes, and both are fused to form a fertilized zygote. The zygote further transforms into a motile ookinete, which penetrates the gut epithelium of mosquito and forms an oocyst. Next, the oocyst undergoes maturation by several rounds of cell division producing numerous sporozoites. Upon oocyst maturation, the sporozoites are released into the mosquito body cavity and invade the salivary glands. The infected mosquitoes, upon a subsequent blood meal, will inject sporozoites into humans thereby marking the beginning of malaria infection in the human host [2, 3]. The parasite encounters major bottlenecks at every stage of its sexual development within the mosquitoes. Several hosts as well as environmental factors are believed to contribute parasite losses within the mosquitoes [4]. The majority of parasite losses are attributed to the action of immune responses mounted by mosquitoes [5]. Among environmental factors, ambient temperature and mosquito diet were found to be important contributors [6]. Additionally, the presence of microbiota in the gut of mosquitoes has gained increased attention in recent years for their ability to modulate *Plasmodium* infection in *Anopheles* mosquitoes [7, 12, 14].

*Anopheles*, like other insects, harbour diverse microbiome in their guts [8]. Several studies have characterized the midgut bacterial diversity in *Anopheles* species using culture-dependent and culture-independent methods [9–11]. Studies have also shown the ability of midgut bacteria from *Anopheles* and non-*Anopheles* mosquitoes to impart negative influence on *Plasmodium* development within mosquitoes [12–14]. The inhibition of *Plasmodium* development within the mosquito by the bacteria is through the production of metabolites, direct interaction with the parasite and/or through induction of mosquito immune responses [14, 15]. In this context, it was found worthwhile to study the microbiota associated with the malaria mosquitoes.

Earlier studies have reported that the microbial composition are associated with the gut of *Anopheles*. A study by Rani et al. [9] identified *Paenibacillaceae* in male and *Serratia marcescens* in female adults as the dominant bacteria in field-collected *An. stephensi* from India. Another study by Chavshin et al. [10] identified 25 Gram-negative species in five genera, including *Pseudomonas*, *Alcaligenes*, *Bordetella*, *Myroides*, and *Aeromonas* in the midgut of *An. stephensi* from southern Iran. Further, Djadid et al. [11] mainly found *Gammaproteobacteria* class, including *Pseudomonas* sp. and *Aeromonas* in the midgut of *An. stephensi* and *Anopheles maculipennis*, respectively from northern and southwestern Iran.

Studies have also shown the impact of midgut bacteria on the development of *Plasmodium* in the mosquito host. The *Enterobacter* bacterium (*Esp*_Z) isolated by Cirimotich et al. [12] from field-caught *Anopheles arabiensis* in Zambia, has been shown to render 99% resistance to *P. falciparum* infections in *Anopheles gambiae*. *Chromobacterium* (*Csp_P*) isolated by Ramirez et al. [13] showed that ingestion of Csp_P by the mosquitoes significantly reduced its susceptibility to *P. falciparum* and dengue virus infections, thereby compromising mosquito vector competence. Bahia et al. [14] found significant inhibition of *P. falciparum* infection in *An. gambiae* by *Pseudomonas putida*, *Pantoea* sp., and *Serratia marcescens* isolated from the midgut of *An. gambiae*. The present study was aimed to characterize the native midgut microbial diversity associated with laboratory-reared mosquitoes, colonized from the progeny of wild-caught mosquitoes from three different geographic locations of southern India (Bengaluru, Chennai and Mangaluru) with varied malaria endemicity; and, to ascertain the influence of total midgut microbiota on the susceptibility of mosquitoes to *Plasmodium berghei* infections.

## Methods

### Mosquito collection and characterization

Immature stages of *An. stephensi* were sampled from their natural aquatic habitats from three cities of southern India, namely Bengaluru, Chennai and Mangaluru using a standard dipping technique. *Anopheles* larvae were separated and identified based on the morphology and distinct mobility pattern, and the rest of the non-anopheline larvae were discarded. The collected specimens, with the breeding site water, were transported and reared in the insectary of National Institute of Malaria Research at Bengaluru. Adult mosquitoes after emergence were identified based on morphological characteristics [16].

### Colonization of mosquitoes in the laboratory

Field-collected mosquitoes were maintained at the standard insectarium condition of 27 ± 1 °C temperature, 70–80% humidity with 12:12 h light and dark cycle [17]. The mosquitoes were allowed to feed on human blood from a donor through an artificial membrane feeding technique for egg production. After 3–4 days post-blood meal, eggs were collected in ovitraps and transferred to the rearing trays containing dechlorinated water for larval emergence. The ecological variants of *An. stephensi* were identified counting the number of ridges on egg floats, and the type forms (true vector) were selected for rearing [18]. Larvae were maintained in the rearing trays at a density of ~ 200 larvae/l of water and fed with a mixture of ground yeast tablet and dog biscuit in a ratio of 60:40. Resulting pupae from days 10–12 onwards were collected daily and kept in mosquito cages for adult emergence. Emerged adults were maintained on sterile 10% glucose solution. Adult female mosquitoes from F6–F8 generations were used for the metagenomics and *Plasmodium* infectivity studies.

### Midgut isolation, DNA extraction, 16S rRNA gene sequencing, and analysis

Midgut samples were prepared from 35 female mosquitoes each from three laboratory-reared strains (Bengaluru, Chennai, Mangaluru). Mosquitoes were surface sterilized with 70% ethanol and rinsed thrice with sterile PBS (phosphate-buffered saline) as described elsewhere [19]. The midguts were isolated aseptically under a stereomicroscope in the laminar hood. The total genomic DNA was isolated from mosquito midgut tissues using GenElute™ bacterial genomic DNA kit (Sigma, USA) according to manufacturer’s instructions and quantified using a NanoDrop™ 2000 spectrophotometer. Further, the third hypervariable (V3) region of 16S ribosomal RNA gene (approx 280 bp) was amplified (PCR1) from 10 ng genomic DNA using specific primers 341F 5′CCTACGGGAGGCAGCAG3′ and 518R 5′ATTACCGCGGCTGCTGG3′ in a PCR thermocycler. The master mix contained 5 µl of 5× Phusion^®^ HF reaction buffer (NEB), 0.4 µl of 10 mM dNTP solution mix (NEB), 0.2 µl of Phusion^®^ HF DNA polymerase (NEB), 2 µl of forward and reverse primers at 10 pM concentration. The total reaction volume was made up to 25 µl with nuclease-free water. PCR reaction was set at 98 °C for 30 s, 30 cycles of 98 °C for 10 s, 72 °C for 30 s, extension at 72 °C for 5 s. PCR products were run on 2% agarose gel with SYBR Safe DNA gel stain. The amplified product was purified using PureLink Quick Gel Extraction and PCR Purification Combo Kit (Invitrogen, USA) according to the manufacturer’s instructions. The DNA concentration was measured by Qubit^®^ 2.0 Fluorometer. A subsequent PCR reaction was performed with a set of primers containing Illumina indexed barcode sequences. NEBNext^®^ Ultra™ DNA Library Prep Kit for Illumina (New England Biolabs, USA) was followed to construct sequencing library according to the manufacturer’s protocol. The PCR master mix contained 10 μl of 5× Phusion^®^ HF reaction buffer (NEB), 1 μl of 40 mM dNTP, 0.4 μl of 2 U/μl F-540 Special Phusion^®^ HS DNA Polymerase (NEB), 2 μl each 10 pmol/μl forward and reverse primers, 10 μl (minimum 5 ng) of PCR1 amplicon. The total reaction volume was made up to 50 µl with nuclease-free water. PCR reaction was set at 98 °C for 30 s, 15 cycles of: 98 °C for 10 s, 72 °C for 30 s, and extension at 72 °C for 5 s. During library preparation, negative control was maintained in the PCR steps by adding nuclease-free water without DNA material to assess external contamination. Since, amplification products were not found in the negative controls, they were excluded from the sequencing and downstream analysis. The PCR cleanup was carried out with PureLink Quick Gel extraction kit (Invitrogen, USA). The library validation was done on Agilent 2200 TapeStation Instrument. The 16S rRNA libraries were sequenced using pair-end (150 bp × 2) method in an Illumina MiSeq instrument with a V2 Illumina MiSeq Kit (Illumina) according to the standard protocol. In the beginning, reads obtained from the sequencer were subjected to quality checks, such as base quality score distributions, average base content per read and GC content in the reads. Further, V3 region from Illumina paired-end sequences were extracted by trimming the spacer and conserved region and a consensus V3 region sequence is constructed using ClustalO program v.1.2.4 with zero mismatch overlap. Before starting the analysis, the following pre-processing steps such as chimera removal by de-novo chimera removal method UCHIME implemented in the tool USEARCH (version 10.0.240) were followed with default parameters. The downstream sequence analysis was performed using QIIME (Quantitative Insights into Microbial Ecology) (version 1.9.1). Reads from all samples were pooled and clustered into Operational Taxonomic Units (OTUs) based on their sequence similarity (similarity cut-off = 0.97) using UCLUST program (version 1.2.21q). Representative sequences were identified for each OTU and aligned against Greengenes (version 13_8) core set of sequences using PyNAST (version 1.2.1) program. As a result of OTU clustering and taxonomy annotation based on Greengenes database, each representative V3 consensus sequences were assigned with taxonomy from phylum to species level and their sample wise abundances were calculated from taxonomy annotation file using in-house PERL program (see Additional file 1). The taxonomy classification was performed using RDP (Ribosomal Database Project) classifier (version 2.2) against SILVA OTUs database (version 119) with uclust similarity search cut-off of 0.8 (considering more than 80% similarity reads) to search up to taxon level 6 (i.e., up to species). The biological observation matrix (BIOM) has been normalized before rarefaction to consider uneven sample counts. Further OTU tables were rarefied by computing alpha diversity metrics using Shannon, Chao1 and observed species method to observe the species richness and evenness (see Additional file 2). The alpha rarefaction calculation was performed using QIIME v.17 software. The relative taxonomy abundance was measured and plotted using R package v3.4.3.

### In vivo maintenance of *Plasmodium berghei* in mice

The asexual blood stages of green florescence protein-expressing transgenic *P. berghei* (strain ANKA) parasites (MR4—Malaria Research and Reference Reagent Resource Center) were established in 6–8 weeks old BALB/c mice by injecting 10^5^ infected red blood cells (RBCs) through intraperitoneal route. The development of blood stage parasites was monitored by examining the Giemsa-stained thin smears prepared with blood collected from the tail vein [20].

### Antibiotic treatments for mosquitoes

Single cohort of 2–3 days old female *An. stephensi* were fed with combination of antibiotics (15 µg/ml gentamicin, and 100 units penicillin, 100 units streptomycin combination) in sterile 10% glucose solution containing 0.05% para-aminobenzoic acid for 3 days to clear the endogenous bacteria present in mosquitoes [14, 21]. Following antibiotic treatment, the mosquitoes were maintained in sterile 10% glucose solution for 24 h to reduce the impact of residual antibiotics. Untreated batches of mosquitoes were fed only with sterile 10% glucose solution containing 0.05% para-aminobenzoic acid. The clearance of gut microbiota from the mosquitoes by the antibiotics was verified utilizing 16S rRNA gene amplification method as described elsewhere [22]. In brief, V3 region of 16S rRNA gene from the mosquito gut microbiota was amplified with bacterial universal primer 27F 5′-AGAGTTTGATCCTGGCTCAG-3′ and 1492r 5′-TACGGCTACCTTGTTACGACTT-3′ [9]. The PCR was run at 95 °C for 5 min followed by 35 cycles of 30 s at 95 °C, 30 s at 55 °C, and 2 min at 72 °C, followed by an extension step of 72 °C for 10 min.

### *Plasmodium berghei* infection in *Anopheles stephensi*

Cohorts of antibiotic-treated and untreated mosquitoes starved for 12 h were allowed to feed on anesthetized mice infected with *P. berghei*-GFP parasite (5–6% parasitaemia, showing 2–4 exflagellation centres per field) for 20 min. The unfed and partially fed mosquitoes were removed with aspirators and discarded leaving only fully engorged ones. The mosquitoes were maintained at a constant temperature of 19 °C with 70–80% RH until the time of dissections. Day 10 post-infectious blood meal, mosquito midguts were carefully isolated and observed under the Fluorescence microscope (Zeiss Axio Imager A1, Germany) and oocyst numbers were recorded [23].

### Statistical analysis

Statistical analysis such as Mann–Whitney test was performed to assess significant differences in the intensity of oocyst infection between antibiotic-treated and untreated batches of mosquitoes using GraphPad Prism^®^ v.5.00 (GraphPad Software Inc, San Diego, CA, USA).

## Results

### *Anopheles* identification

The mosquitoes collected from three cities of southern India, Bengaluru, Chennai and Mangaluru were identified as *An. stephensi* type form based on their morphological characteristics and egg ridge numbers [16–18].

### Bacterial diversity associated with the midgut of three laboratory-reared strains of *Anopheles stephensi*

**Fig. 1:**
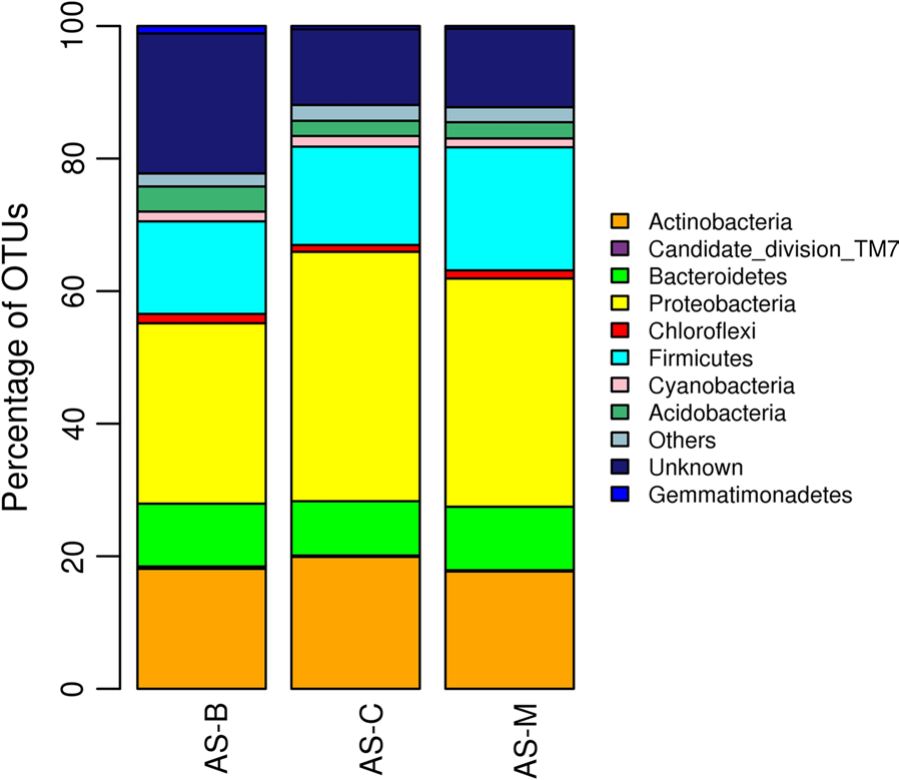
Taxonomy classification of Operational Taxonomic Units (OTUs) at the phylum level for the midgut sample of *Anopheles stephensi* strains derived from Bengaluru (AS-B), Chennai (AS-C), and Mangaluru (AS-M). Only the top 10 enriched class categories are shown

**Fig. 2:**
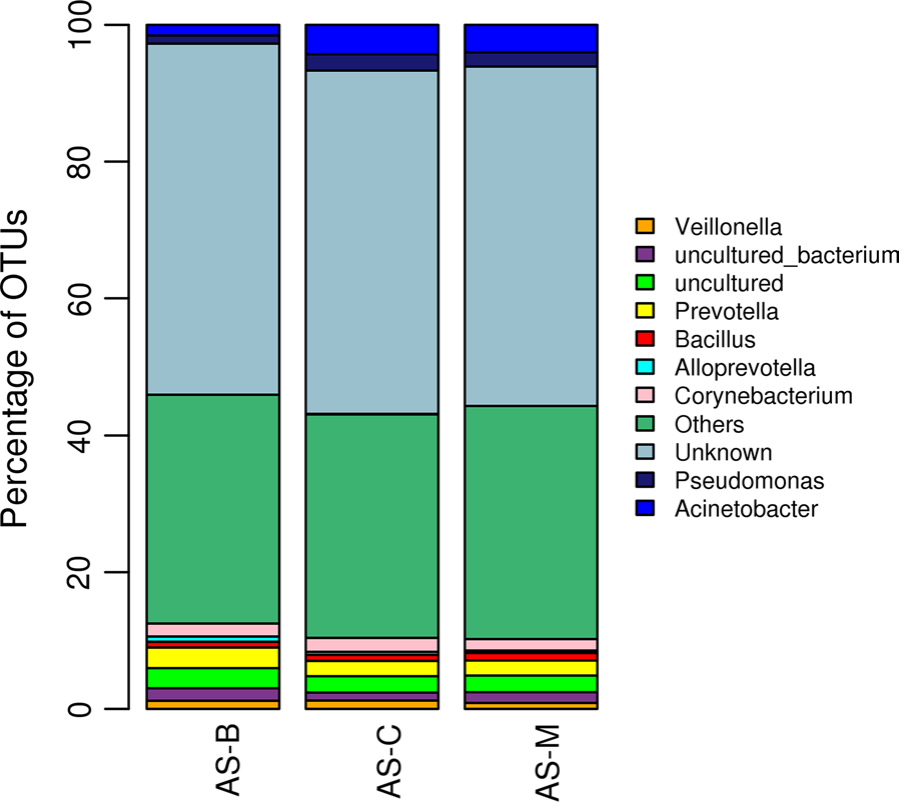
Taxonomy classification of Operational Taxonomic Units (OTUs) at the genus level for the midgut sample of *Anopheles stephensi* strains derived from Bengaluru (AS-B), Chennai (AS-C), and Mangaluru (AS-M). Only the top 10 enriched class categories are shown

Midgut bacterial diversity analysis of three laboratory-reared strains of *An. stephensi* was carried out by 16S rRNA gene sequencing approach. The sequencing of V3 region of 16S rRNA gene by Illumina Miseq platform generated 813,827; 867,769 and 915,719 total reads from *An. stephensi* midgut samples from Bengaluru (AS-B), Chennai (AS-C) and Mangaluru (AS-M) strains, respectively. After quality control, a total of 651,490 reads were considered, from which 2944 OTUs were picked. The midgut bacteria analyses among the three laboratory-reared strains of *An. stephensi* were found to be quite similar. Most abundant OTUs were associated with phylum *Proteobacteria* with 27.2%, 37.6 and 34.4 in AS-B, AS-C and AS-M strains, respectively. Other observed abundant phyla found in all field-derived strains of mosquitoes were *Actinobacteria*, *Firmicutes*, *Bacteroidetes*, *Acidobacteria*, *Cyanobacteria*, and *Chloroflexi* (Figs. 1, 2).

### *Plasmodium berghei* infection status in three field-derived strains of *An. stephensi* under the influence of total gut microbiota

**Fig. 3:**
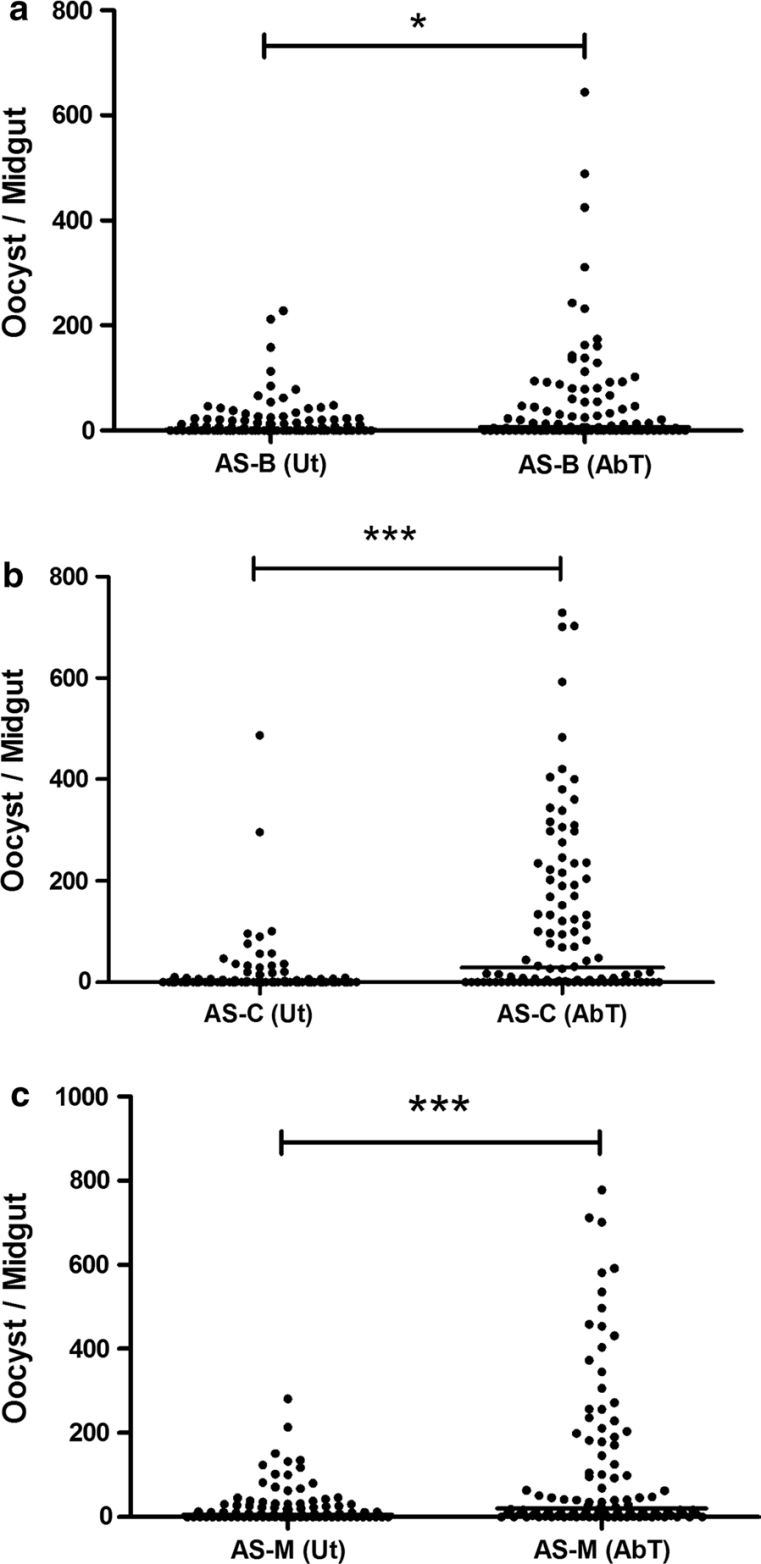
*Plasmodium berghei* oocyst intensity in antibiotic-treated (AbT) and untreated (Ut) batches of field-derived *Anopheles stephensi* (AS) strains from **a** Bengaluru [AS-B (AbT)] and [AS-B (Ut)], **b** Chennai [AS-C (AbT)] and [AS-C (Ut)], **c** Mangaluru [AS-M (AbT)] and [AS-M (Ut)]. The significant differences between oocyst intensity between AbT and Ut batches were assessed by Mann–Whitney test (*P < 0.05; ***P < 0.001). (n = 100/group). Circles indicate the number of parasites from an individual mosquito, and horizontal lines indicate the median

**Fig. 4:**
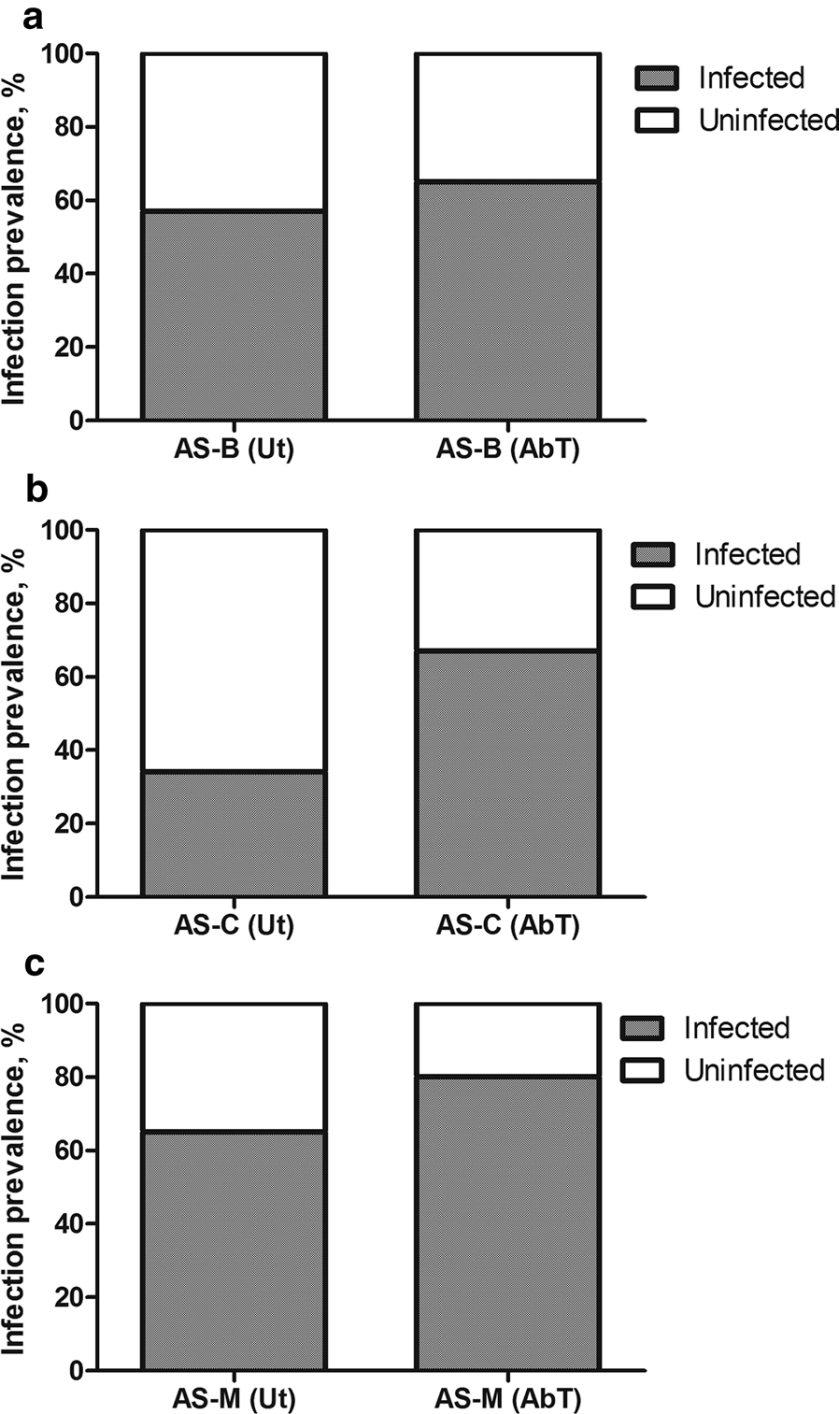
Prevalence of oocyst in *Plasmodium berghei*-infected, antibiotic-treated (AbT) and untreated (Ut) batches of *Anopheles stephensi* (AS) strain from **a** Bengaluru [AS-B (AbT)] and [AS-B (Ut)], **b** Chennai [AS-C (AbT)] and [AS-C (Ut)], and **c** Mangaluru [AS-M (AbT)] and [AS-M (Ut)]

**Fig. 5:**
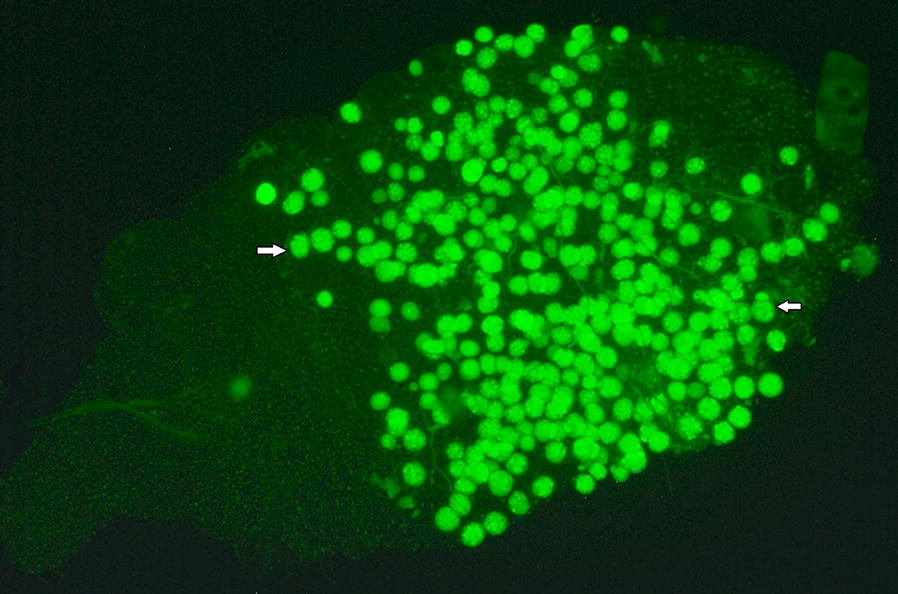
*Plasmodium berghei* (PbGFP) oocysts in the midgut of *Anopheles stephensi*

The method to verify the clearance of bacteria from the gut of antibiotic-treated mosquitoes by PCR amplification of 16S rRNA gene by bacteria universal primer showed no or minimal amplification, whereas in untreated females substantial amplifications were observed. The influence of total midgut microbiota of *An. stephensi* on *P. berghei* development was studied by comparing the intensity (number of oocyst/mosquito) and prevalence (percentage of mosquitoes infected) of *Plasmodium* infection among cohorts of mosquitoes treated with antibiotics to remove their native midgut bacterial flora with antibiotic-untreated mosquitoes containing its natural native microflora. A significant difference in *P. berghei* oocyst intensity was observed among antibiotic (AbT)-treated and untreated (Ut) batches of *An. stephensi* laboratory strains from Bengaluru (P < 0.05), Chennai (P < 0.001) and Mangaluru (P < 0.001), where the oocyst load in the AbT batch was found to be higher compared with Ut in all three tested mosquito strains (Fig. 3). The mean numbers of oocyst per midgut in AbT and Ut batches of *An. stephensi* strain derived from Bengaluru were 51.07 and 18.48, respectively, that from Chennai 121.7 and 16.4, respectively, and from Mangaluru 115.1 and 25.39, respectively. Furthermore, the prevalence of *P. berghei* infection in *An. stephensi* was found to be higher in AbT batches of mosquitoes compared with Ut batches in all three tested mosquito strains (Fig. 4). The *P. berghei*-infected *An. stephensi* gut containing GFP-expressing oocysts is shown in Fig. 5. Lower prevalence and intensity of infection in Ut batches of mosquitoes compared with AbT ones clearly indicates the negative influence of total midgut microflora on *P. berghei* development in *An. stephensi*.

## Discussion

The midgut bacterial diversity study of laboratory-reared strains of *An. stephensi* identified *Proteobacteria* as the dominant phyla. Rani et al. [9] made similar observation where *Proteobacteria* was the dominant phyla in the midgut of field-collected *An. stephensi* mosquitoes from Haryana, India. *Proteobacteria* was identified as dominant phyla among field-collected *An. stephensi* and *An. maculipennis* from Iran, *An. gambiae* from Cameroon [11, 24]. Ngo et al. also found *Proteobacteria* as the dominant phyla in several of *Anopheles* species sampled from two different regions of Vietnam, Dak Nong and Binh Phuoc Provinces [25, 26]. Most of the dominant genera in the midgut of *An. stephensi* identified here, namely *Acinetobacter*, *Pseudomonas*, *Bacillus*, *Prevotella*, and *Corynebacterium*, have been previously reported in *Anopheles* mosquitoes [8, 9]. Interestingly, *Veillonella* genera were found in the midgut of *An. stephensi* for the first time, and reported in the present study. However, the presence of *Veillonella* sp. has been reported by Fraihi et al. [27] in the midgut of laboratory-reared sand fly (*Phlebotomus perniciosus*, Diptera: Psychodidae). Also, McCarthy et al. [28] found uncultured *Veillonella* sp. in *Lutzomyia longipalpis*, a vector of visceral leishmaniasis. In general, the midgut microbial composition in mosquitoes is found to be influenced by various factors, such as species of mosquitoes, life stages, sex, feeding behaviour, geographical locations, seasonality, etc. [29, 30]. In contrast to other studies, a homogenous microbial population among laboratory-reared stains of *An. stephensi* mosquitoes was found in the present study, which may be due to the fact that all the mosquito strains were grown under identical conditions with the same diet for 6–8 generations before sampling them for bacterial diversity studies [24, 30]. Studying the microbial communities through 16S rRNA gene sequencing is subject to many pitfalls and many sources of bias have been identified with sample processing steps [31]. Importantly, work up of negative controls alongside samples throughout has been found to be very critical to assess possible contaminants from kits and reagents [32]. Here, not sequencing the negative controls because of undetectable library yields and excluding the reads from them in downstream analysis might have increased the chances of introducing contaminants thereby misleading the results, so that counteracting bias in metagenomic study is found to be very critical. A study by Boissière et al. [24] found significant correlation between *Enterobacteriaceae* abundance in the mosquito midgut with *Plasmodium* infection status. The present study also recorded *Enterobacteriaceae* in all field-derived *An. stephensi* at an OTU percentage of 1.8% in AS-B, 2.0% in AS-C and 1.4% in AS-M strains. Some species from the genus *Pseudomonas* are among *Anopheles* gut-derived bacteria with known *Plasmodium*-blocking abilities [14, 33]. *Pseudomonas* genera in the midgut of all field-derived strains of mosquitoes were detected in the present study. Most of the studies have reported the effect of individual bacteria isolates from the gut of mosquitoes on *Plasmodium* development in its host [12–14]. An effort was made to understand the effect of total or entire mosquito midgut microbiota on susceptibility of *An. stephensi* originally from 3 different locations to *Plasmodium* infections, as this could recognize the influence of entire microbiota on host vector competence [24]. Although, the lower *Plasmodium* infection level among the three tested *An. stephensi* strains in the presence of total microbiota was observed, a more comprehensive study is required to draw reliable conclusions on the relationship between the difference in microbiota composition and variation in competence of mosquito for *Plasmodium* infections. In recent years, researchers have shown increased emphasis on the use of midgut microbiota of vector mosquitoes in control of vector-borne disease [34, 35]. In this regard, understanding the diversity, function and dynamics of midgut microflora of *Anopheles* would offer newer prospective in control of malaria transmission.

## Conclusions

Among the newer tools to control vector-borne diseases, the use of microbial symbionts to reduce vector competence has gained much importance. The current study was designed to identify the midgut microbiota composition of *An. stephensi*, and to identify the effect of total mosquito-microbiota on the development of rodent malaria parasite in *An. stephensi*–*P. berghei* model. Microbial survey in *An. stephensi* midguts revealed *Proteobacteria* as the dominant phyla in the midguts. The association of genus *Veillonella* with *Anopheles* was revealed for the first time reported in the present study. Further, the infectivity studies in mosquitoes clearly showed that the total microbiota of the mosquitoes can negatively influence parasite development thereby reducing the competence of mosquitoes to disease transmission. Therefore, it is important to understand the native microbiota composition of vectors and its relationship with disease transmission in the context of designing transmission blocking approaches to control the disease.

## Additional files

**Additional file 1.** The Greengene final annotation summary contains OTU ids, cumulative V3 read count, sample-wise read count, consensus V3 sequence fasta sequence, taxonomy representation from phylum to species level.

**Additional file 2.** Alpha diversity with samples and rarefaction curves; Beta diversity between samples.
