# Supplementary material for: Influence of midgut microbiota in Anopheles stephensi on Plasmodium berghei infections
Source: Malar J. 2018 Oct 25;17:385. doi: 10.1186/s12936-018-2535-7 (PMC6203276; doi:10.1186/s12936-018-2535-7)
Supplement: Supplementary file 2 — Additional file 2. Alpha diversity with samples and rarefaction curves; Beta diversity between samples. [file 12936_2018_2535_MOESM2_ESM.doc]

# Alpha diversity with samples and rarefaction curves

Here, the microbial diversity within the samples was analyzed by calculating Shannon, Chao1 and observed species metrics. The species richness and evenness among samples was calculated using “alpha_diversity.py” program available in QIIME software. The chao1 metric estimates the species richness, while Shannon metric is the measure to estimate observed OTU abundances, and accounts for both richness and evenness. The observed species metric is the count of unique OTUs identified in the sample. The rarefaction curve for each of the metric is provided in Figure.1-3. The metric calculation was performed using QIIME software.

The sample AS-C was having rarefaction index value of 5.5 whereas AS-M with 4.5 and AS-B with 4.2. This indicates that AS-C was distributed with more unique OTUs each corresponding to unique species.


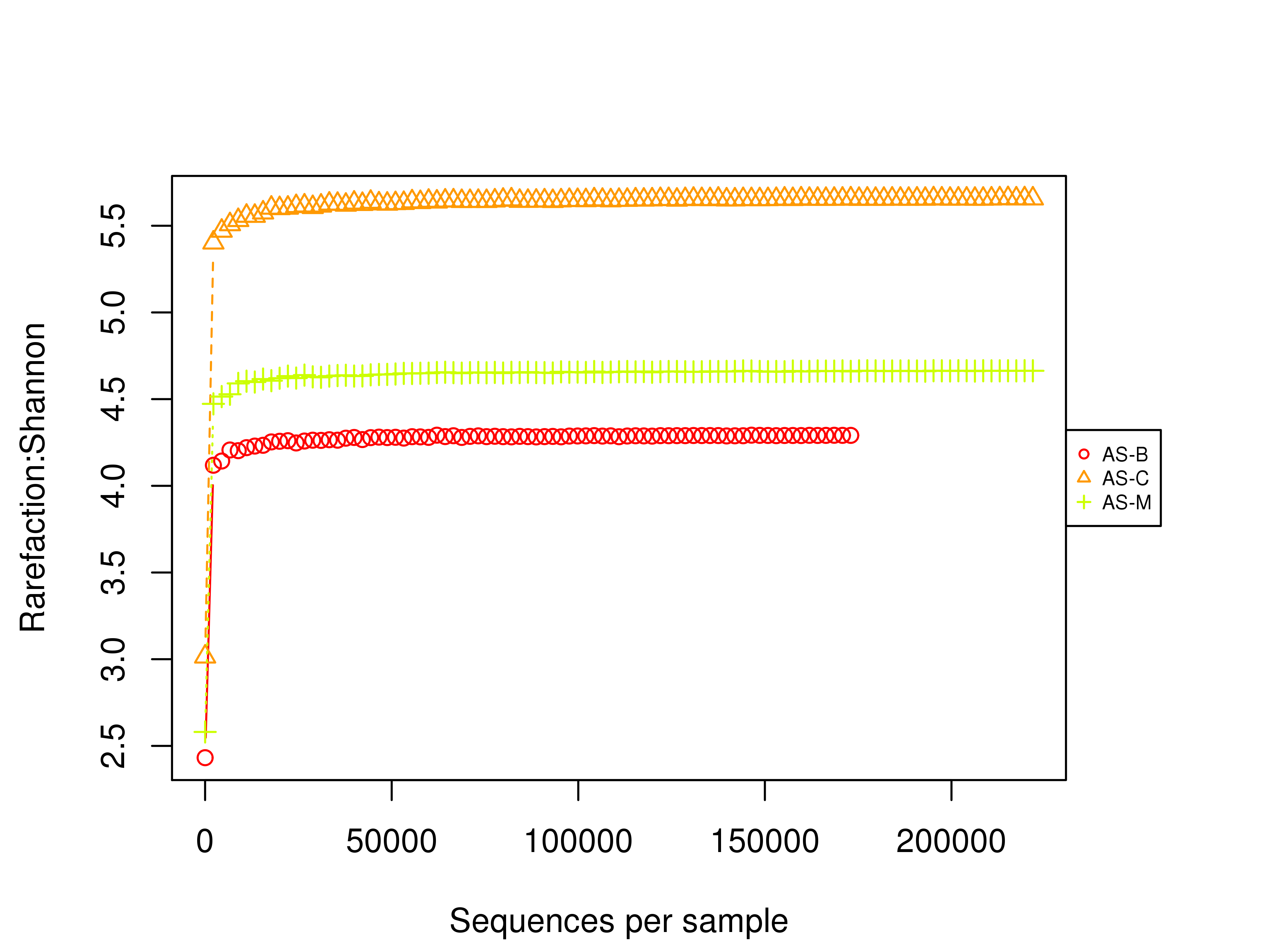


**Figure S1.** Shannon curve obtained for the samples. Alpha diversity was computed using Shannon metrics with rarefied OTU table size of 100.

**
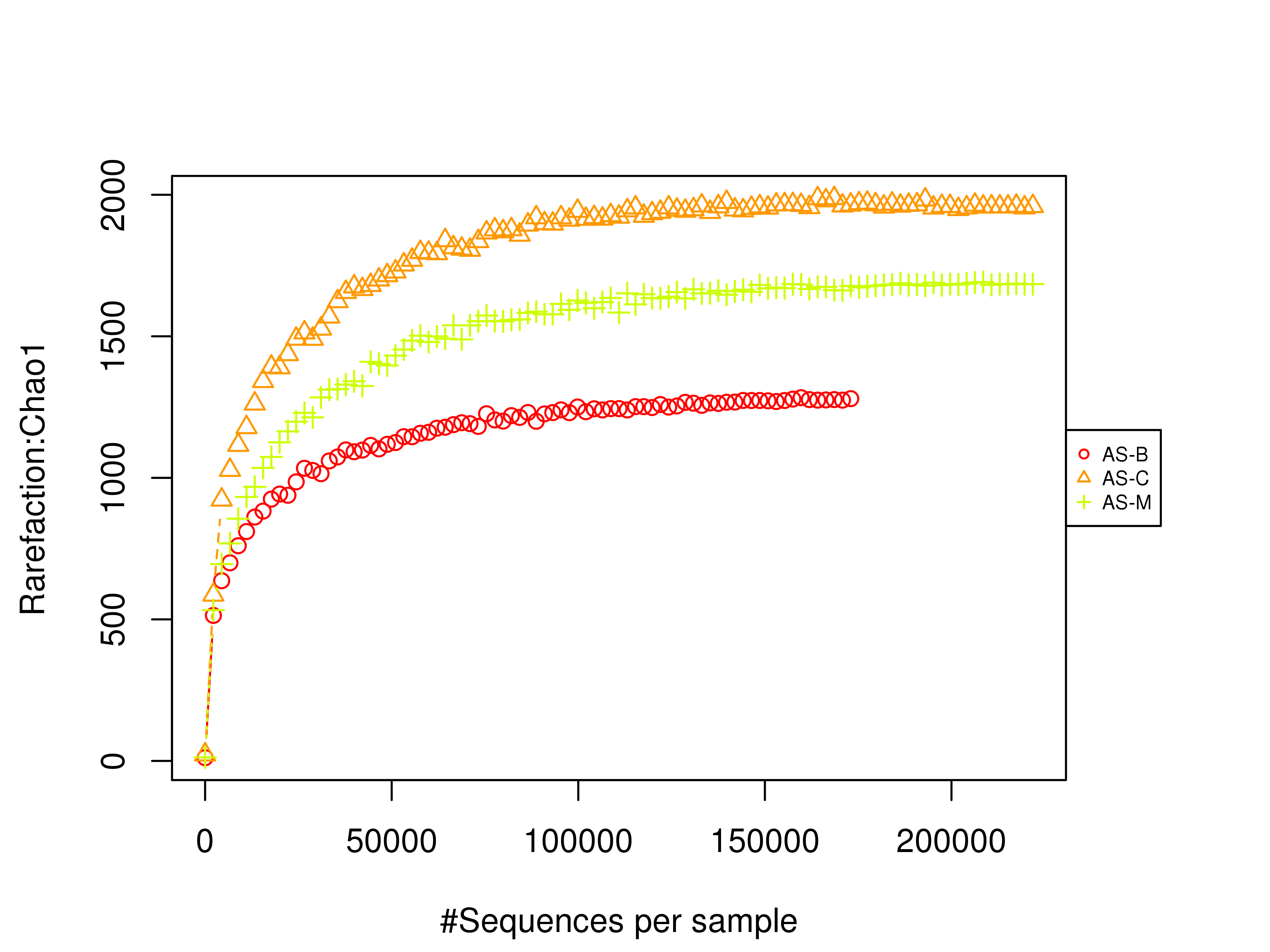
**

**Figure S2.** Chao1 curve obtained for the samples. Alpha diversity was computed using Chao1 metrics with rarefied OTU table size of 100.


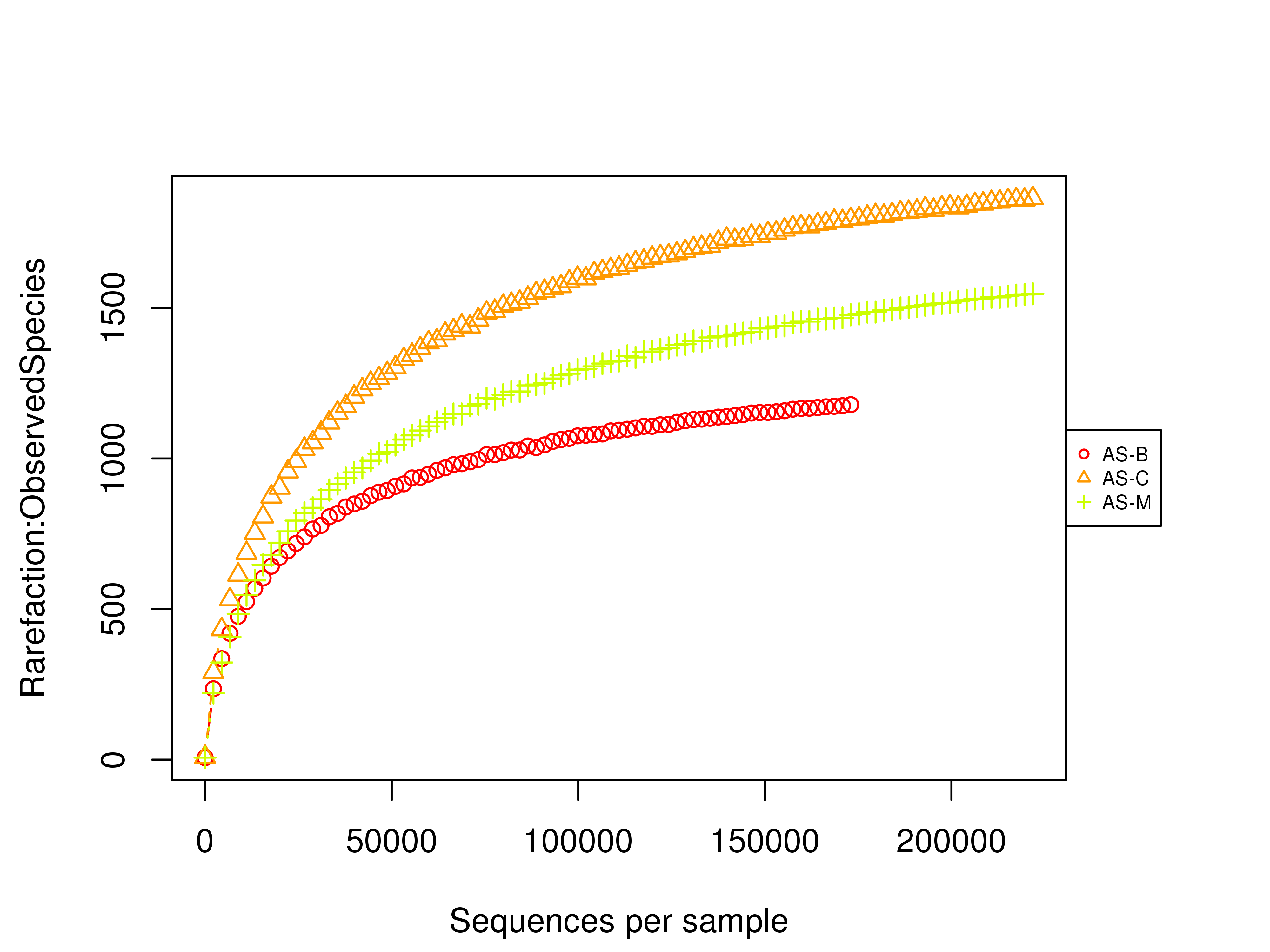


**Figure S3.** Observed species curve obtained for the samples. Alpha diversity was computed using observed species metrics with rarefied OTU table size of 100.

# Beta diversity between samples

In this section, explicit comparison of microbial communities between the samples was compared. At the beginning, we generated the distance matrix using weighted UniFrac approach. Sequence abundances were taken in to account in Weighted UniFrac for comparing microbial diversity. A jackknife test was performed to construct a consensus UPGMA (Unweighted Pair Group Method with Arithmetic Mean) tree for all samples. The resulted consensus UPGMA trees built using weighted ddistance matrix is shown in Figure.4.


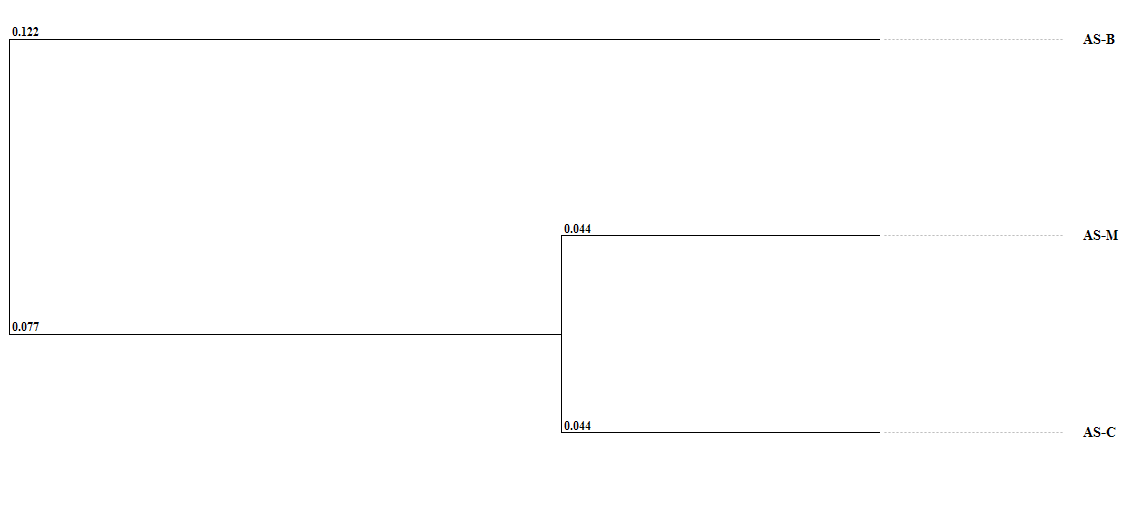


**Figure S4.** Phylogenetic tree based on weighted unifrac approach.
